# Longitudinal Characteristics of Glioblastoma in Genome-Wide Studies

**DOI:** 10.1007/s12253-019-00705-1

**Published:** 2019-08-03

**Authors:** Zoltan Kraboth, Bernadette Kalman

**Affiliations:** 1 Graduate School in Neurosciences, University of Pecs, 12. Szigeti street, Pecs, 7624 Hungary; 2 Institute of Laboratory Medicine, University of Pecs, 13. Ifjusag street, Pecs, 7624 Hungary; 3 Szentagothai Research Center, University of Pecs, 20. Ifjusag street, Pecs, 7624 Hungary

**Keywords:** Glioblastoma, Sequential samples, Molecular evolution, Genomics, Transcriptomics, Epigenomics

## Abstract

Glioblastoma is one of the deadliest tumors with barely over one-year median survival despite intensive efforts in defining its molecular characteristics and searching for innovative treatment strategies. While major progress has been made in cataloging cross-sectional genomic, transcriptomic and epigenomic features of the tumor, and inferring its main molecular pathways and niches for potential targeted intervention, we still do not have sufficient knowledge concerning evolutionary patterns and dynamics of molecular changes or the treatment-induced effects affecting glioblastoma biology. In this review, we summarize the results of recent longitudinal genomic, transcriptomic and epigenomic studies that brought us closer to a better understanding of this lethal disease. Evidence suggests that neuronal / glioma stem cells with accumulating mutations initiate glioblastoma development and recurrence, but the hypothetical models describing the courses that lead to established tumors have not been fully proven. Moving from the histopathological phenotype to the results of high resolution OMICS studies, we try to synthesize the currently available information from sequential glioblastoma analyses in order to highlight its multifaceted features and heterogenetity, as well as the expected complexity of potential treatment strategies that might once succeed.

## Introduction

Glioblastoma (GBM), a grade IV glioma according to the classification by the World Health Organization [WHO], is one of the most common, and most malignant brain tumors. The majority, 90% of GBM, is primary tumor arising de novo*,* while the remaining subset is secondary that progresses from a WHO grade II diffuse astrocytoma or WHO grade III anaplastic astrocytoma [1].

Characteristically, GMB recurs usually within a year despite aggressive therapy [2]. The standart treatment of newly diagnosed GBM involves radiotherapy and temozolomide (TMZ)-based chemotherapy following gross total resection [3]. These interventions somewhat slow the progression of the tumor, but inevitably, a small residual population of cells escapes surgery and chemoradiation, and results in a typically fatal recurrence in about 7 months after diagnosis [4]. In contrast to the initially diagnosed tumors, there is no standard treatment protocol for the recurrent GBMs as yet. With the currently avaiblable medications, the median overall survival time is 12–15 months from initial diagnosis, although with the improvements of interventions and supportive care the figure may somewhat exceed 20 months at certain centers [5, 6]. Identification of the molecular drivers of tumor evolution is of major importance, since a better understandig of the etiology and recurrence might provide clues for the developement of efficient and targeted treatments [7]. The biggest step towards achieving this goal was the genome-wide analyses of chromosomal structural variations, single nucleotide variations (SNVs) and copy number variations (CNVs) in GBM by The Cancer Genome Atlas Network (TCGA) [8]. Subsequently, integrated analyses of genomic and transcriptomic data revealed that GBM may be subdiveded into molecular subtypes named proneural (PN), classical (CL), mesenchymal (MES) and neural (NE) (the latter category was later abandoned, since the pattern turned out to be due to normal cell contamination in the investigated sample) [9, 10]. Parallel, explorations of DNA CpG island methylation patterns genome-wide and correlations of these epigenomic patterns with the molecular GBM subtypes were reported [11]. Our group performed translational studies that reproduced separation of the three (PN, CL, MES) GBM molecular subgroups in formalin-fixed, paraffin-embedded (FFPE) specimens using immunohistochemistry (IHC) and pyrosequencing, and thereby demonstrated that the TCGA OMICS observations, may be applied, at least in part, in clinical practice [8, 9, 12]. The TCGA studies also revealed that the molecular abnormalities in GBM preferentially align in certain pathways including the activation of the epidermal growth factor receptor (EGFR) along with other tyrosin kinase receptors-initiated signaling pathways, the tumor protein 53 (TP53) and retinoblastoma (RB1) pathways [13–17]. These genetic alterations and pathways are known to promote tumor growth, while the roles of several novel or passanger mutations in GBM pathogenesis remain unclear [18]. Recent genomic and transcriptomic studies of sequential samples have also provided important observations for the elucidation of GBM development [4, 7, 10, 18–21]. Thus far, however, there has only been a few studies focused on epigenomic examination of GBM, and particularly, in sequential specimens [22–25]. Nevertheless, these few studies established that, in addition to alterations in the genome, epigenetic modifications, particularly CpG island methylation changes, underly the observed changes in the transcriptome and play an important role in defining disease progression. In depth mapping of genomic, transcriptomic and epigenomic alterations during the evolution of GBM is very important in order to gain reliable insights into tumorigenesis, progression and recurrence, as well as to assist the development of targeted treatments [26].

GBM is a notoriously heterogeneous tumor at both the histopathological and the molecular levels. Great degrees of variability may be observed among and within GBMs regarding grade as well as clonality. Intratumor heterogeneity (ITH) may reflect simultaneous presence of gliomas of various grades or GBM of various molecular subtypes, although the latter has been the subject of some debate. Similarly, whether GBM retains its original molecular subtype over time, or different transcriptional subtypes may be present at diagnosis and relapse has not been unequivocally established [8, 27, 28]. Evidence suggests that mutations accumulating in neuronal/glioma stem cells initiate the development and contribute to the recurrence of glioblastoma (for details see *Tompa* et al. [29]*)*. Two common models have been proposed for capturing how heterogeneity arises and therapy resistance develops during the tumor’s course. The first model includes the cancer stem cell hypothesis or the ancestral cell origin model [30] assuming that a relatively small subset of cells with stem cell-like properties give rise to different tumor cell populations. In these cells, resistance develops through the acquisition of novel genetic alterations (GA). In contrast, the alternative clonal evolution model [31] suggests that all tumor cells independently acquire novel GA and later undergo natural selection. Every subclonal population has the potential to expand as enviromental or therapeutic conditions change [4, 18]. Which one of these two models describes tumor evolution more accurately remains to be determined. The aim of this survey is to provide a brief overview of genomic, transcriptomic and epigenomic data obtained from progressive GBM tumors in order to highlight main features and dynamics of tumor evolution.

## Comparative Profiles of Initial and Recurrent GBMs (Table 1)

While reports on molecular characterization of GBM represent a significant proportion of the scientific literature, there are relatively few studies comparing the molecular profiles in primary and recurrent tumor pairs. There are two main reasons for this scarcity of information. First, only 25% of recurrent tumors are amenable to surgery, which leads to a very restricted number of suitable samples [5]. Second, even if the recurrent GBM tissue can be removed, the extent of necrosis, that is typically much more extensive in the recurrent tumor, often prohibits its use in further molecular studies [32]. However, because of the importance of information gained from such sequential profiling, we summarize below the available data, first presenting observations relying on selected markers, and then those relying on genome-wide analyses.

**Table 1:** Summary of main findings in longitudinal studies

	Study	Paired samples (n)	Experimental methods	Main findings
Selected markers	Stark et al. 2003 [3]	27	IHC	- MLH1, MSH2, TP53 expression significantly lower in recurrent tumors
	Shinsato et al. 2013 [33]	11	IHC	- MLH1, PMS2 protein levels are reduced in TMZ-resistant cells - MLH1 induced PMS2 protein instability confers TMZ resistance to GBM cells
Genomic and transcriptomic analyses	Kim J. et al. 2015 [4]	38	WES, RNA-seq	- Distally recurred tumors, in contrast to locally recurred ones, share only a minority of initial tumor mutations - Primary GBMs rarely develop hypermutation after TMZ treatment
	Wang J. et al. 2016 [19]	114	WES, Transcriptome analysis	- 2/3 of primary GBMs switched subtype upon recurrence - Hypermutation preferentially targets highly expressed genes - Novel mutation in LTBP4 found in 11% of recurrent tumors - TGF-β pathway is a potential therapeutic target
	Martinez et al. 2009 [7]	20	Semiquantitative PCR, LOH analysis	- Initial tumors without P53 and EGFR mutations acquired new EGFR amplification upon recurrence - Recurrences display two distinct patterns depending on the profile of the original tumor
	Sottoriva et al. 2018 [43]	11	WES, TES	- Multi-regional WES revealed extensive ITH involving EGRF amplification and the loss of chromosome 10 containing PTEN and CDKN2A
	Kim H. et al. 2019	23	WGS, WES, PyClone clustering	- Mutation clustering seen as clonal (67.9%), subclonal (29.8%) - 90% of TP53 and PIK3CA/PIK3R1 mutations are clonal - TP53 mutational status has influence on clonal tumor progression
	Muscat et al. 2018 [21]	21	WGS, WES, Targeted deep sequencing	- Variant burden reduced in recurrent tumors - Neutral tumor evolution in untreated GBM shifted towards non-neutral evolutionary dynamics in recurrent GBM after treatment - Increased mutation rate occurred in one recurrent tumor, attributable to TZM-induced hypermutator phenotype
	Wang Q. et al. 2017 [10]	8	Transcriptome profiling	- Macrophage/microglia-rich microenvironment shapes the MES phenotype - NF1 deactivation results in macrophage/microglia attraction - Gene-expression subtype retained in 55% of the cases
Epigenomic analyses	Hegi et al. 2005 [22]	206	Methylation-specific polymerase-chain-reaction	- MGMT promoter was methylated in 45% of cases - MGMT promoter methylation was an independent favorable prognostic factor - MGMT promoter methylation results in survival benefit
	de Souza et al. 2018 [24]	77	Comprehensive DNA-methylation analysis	- Classification of diffuse IDH mutant and IDH-wt gliomas - G-CIMP-high subgroup identified with worst prognosis, and the capability to recur as a more aggressive tumor - Predictive biomarkers for assessing the risk of recurrence identified
	Klughammer et al. 2018 [25]	112	RRBS, RNA-seq	- Optimized RRBS can be used to infer transcriptional subtypes - DNA methylation can be predictive of immune cell infiltration, the extent of necrosis and subcellular tumor cell morphology - Recurrent progression-associated demethylation of Wnt promoters in association with worse prognosis

The table highlights observations from analyses with selected markers and from genomic, transcriptomic and epigenomic studies on sequential glioblastoma specimens

## Protein Expression-Based Studies with Selected Markers

IHC is one of the most widely used techniques in the clinical setting and in translational research. It not only helps to establish the histological diagnosis, but also shows the tissue distribution and subcellular localization of expressed proteins. In addition, IHC is suitable for a comparative and quantitative determination of marker expression in various tissue regions. Two previous studies by Stark et al. [33, 34] worked with large numbers of paired samples using IHC, and focused on the expressions of pre-selected proteins with putative roles in DNA repair and tumor growth (MLH1, MSH2, MSH6, TP53 mutation, EGFR amplification). The authors found that expressions of MutL homolog 1 (MLH1), MutS homolog 2 (MSH2) and tumor protein 53 (TP53) (products of DNA repair and tumor suppressor genes) were significantly lower in recurrent tumors. Furthermore, expression levels of MLH1 and MSH2 (and of MSH6 only in initial tumors) were significantly associated with the Ki67 proliferation index in both initial and recurrent tumors, indicating a potential role of these proteins in GBM progression. Similar studies were performed by Shinsato et al. [35] using IHC analyses to compare MLH1, postmeiotic segregation increased 2 (PMS2) and O^6^-methylguanine DNA methyltransferase (MGMT) expression levels in primary and recurrent GBMs, the latter obtained from patients after TMZ treatment. This study also revealed a reduction in the MLH1 and PMS2 protein levels in TMZ-resistant cells. These observations suggest that a reduction in MLH1 protein expression leads to PMS2 protein instability, which in turn, confers TMZ resistance to GBM cells. Although the results were very exciting and the method of IHC could allow for addressing, the above studies did not analyze ITH. In one of our recent translational studies, we also included a small sequential cohort and found that molecular subgroups were largely retained in recurrent compared to primary GBM specimens, however, even with the few markers used in IHC signs of ITH and clonal evolution could be revealed in consensus with much more comprehensive analyses by the TCGA [8, 9, 12]. While these IHC-based studies provided some important observations and prognostic markers, altogether, the methodological approach involved technical limitations with low resolution of the gained information. Therefore, more comprehensive approaches were needed to advance further the field and to gain deeper insights into GBM pathogenesis.

## Overview of Longitudinal Genome-Wide Studies

OMICS methods have been gaining ground since next generation sequencing (NGS) and microarray analyses became available [36]. These high resolution and high throughput methods have provided invaluable information about variations accumulating in the genomic DNA, the transcriptome and epigenome within tumors, including GBM. NGS analyses of genomic DNA define SNV, small insertions / deletions and CNVs genome-wide. It may also provide information concerning chromosomal rearrangements and even chromotripsis (see below). Whole-exome sequencing (WES) and targeted-exome sequencing (TES) reveal similar data, but restricted to the exomes or to a set of selected genes.

Transcriptome studies quantitatively define messenger (m)RNA expression genome-wide typically in bulk RNA of a selected tissue, while single cell RNA analyses are recently becoming more widely used [36]. As a transcriptome captures a snapshot of total transcripts present at a time, transcriptome analyses may allow us to define which cellular processes were active (which genes were expressed) and which were dormant (which genes were not expressed) at that moment in a given specimen. The active genes may align in certain pathways signifying certain biological processes. Mapping gene expression in a tissue (i.e. GBM vs. normal brain tissue) at different time points (at diagnoses and at relapse) provides information about how genes are regulated during the development of the disease.

Epigenetic mechanisms involving enzymatic modifications of DNA and associated histone proteins or altered expression of microRNAs regulate gene transcription and translation. Epigenomic alterations are increasingly recognized as a source of phenotypic variability [37]. DNA CpG island methylation is the most widely studied epigenetic mechanism in GBM, which results from the addition of a methyl group to cytosine to become 5-methylcytosine. This modification is generally observed at 5’-CpG-3′ dinucleotides in all mammals (occasionally observed at CpNpGs) [38], and is required for silencing of genes [39] and allele-specific imprinting [40]. The other important mechanism is the post-translational modification of N-terminal tails of histone proteins by acetylation, methylation, phosphorylation, and other biochemical modifications [41]. Expression changes in the microRNAs have recently been explored in the development and progression of several tumors. These small non-coding RNAs inhibit the translation and stability of mRNAs, and thereby are involved in different cellular processes such as cell cycle regulation, differentiation, apoptosis and migration [42].

GBMs are characterized by alterations affecting genes that control cell growth, apoptosis, angiogenesis and invasion. Genomic, transcriptomic and epigenomic alterations all contribute to these biological processes, and evolve during progression of GBM.

### Genomic Analyses

Using WES and TES of 38 corresponding primary and recurrent GBM pairs, the study by *Kim J.* et al. [20] revealed that distally recurred tumors only shared a minority of initial tumor mutations (25%) indicative of divergent evolution, while most of the locally recurred tumors shared a majority (70%) of initial tumor mutations consistent with linear evolution. These results suggest that although GBMs may recur throughout the brain, recurrence at a distant cerebral location involves a high degree of clonal selection and consequent genomic divergence. The authors also speculate, that GBM clones that had diffusely invaded the brain parenchyma at early stages of tumor developement may wake up from their dormant state and repopulate distant locations with actively growing daughter cells [20]. To further investigate the nature of tumor evolution, another group of investigators carried out the largest-scale sequential study to date, with 114 patients’ samples. In 93 cases, DNA specimens from normal tissues (i.e. normal germ-line DNA sequences) were available in addition to those of primary and recurrent tumor samples. WES and transcriptome analyses showed that although 45% of the mutations are being shared between the primary and recurrent tumors, the dominant clone at diagnosis is generally not a linear ancestor of the dominant clone at relapse [19]. Another important finding in this study revealed that hypermutations preferentially target highly expressed genes, suggesting that the mutagenic mechanisms related to the alkylating effect of TMZ treatment affects most efficiently highly expressed regions of open chromatin. *Wang J* et al. [19] also stated that two-thirds of patients with primary GBM exhibit different transcriptional subtypes at diagnoses and relapse, in contrast to the conclusions of other studies suggesting that GBMs largely retain their initial transcriptional molecular subtypes [9, 10, 12].

A study by *Martinez* et al. [7] analyzed 20 paired GBM samples. Among primary GBMs, the authors observed 4 type 1 GBMs (secondary) which contained p53 mutation without EGFR amplification, 12 type 2 GBMs (de novo) which contained EGFR amplification in the absence of p53 mutation and 4 non-type 1-non-type 2 GBMs in which neither EGFR amplification nor p53 mutation was observed. Interestingly, all type 1 and type 2 GBMs conserved their p53 mutational status and EGFR amplification at relapse. This stability of the EGFR amplification status over time in the majority of the tumors was subsequently confirmed [17]. However, the study by *Martinez* et al [7] also showed that the four non-type 1-non-type 2 GBMs developed new EGFR amplifications, and thus acquired the type 2 GBM profile. These observations suggest that during relapses GBMs may accumulate additional molecular alterations and evolve along two distinct pathways acquiring type 1 GBM profile (harboring p53 mutation) or type 2 GBM profile (harboring EGFR amplification), depending on the profile of the original tumor [7]. The accumulation of new molecular alterations may explain, at least in part, the observed transcriptional subtype switching between initial and recurrent tumors.

A study by *Sottoriva* et al. [43] draws attention to that the residual disease, and not just the main tumor mass, must be investigated to understand how treatment-resistance develops. The investigators obtained multiple samples from multiple regions, including the main tumor mass, the infiltrating margin and the sub-ventricular zone of 11 patients along with their matching blood samples. The multi-regional WES analyses revealed extensive ITH as reflected by SNVs and CNVs, and involved EGFR amplification, the loss of chromosome 10 containing PTEN and homozygous deletions of CDKNA2. The phylogenetic trees built from WES data showed that residual disease subclones can arise early during tumor growth, and these infiltrative subclones may seed the growth of a recurrent tumor after treatment [43]. The genomic road map that leads to recurrence can be highly idiosyncratic, but may broadly be classified into two patterns: 1. linear recurrences share extensive genetic similarity with the primary tumor and can be directly traced to one of its specific sectors, and 2. divergent recurrences that share only few genetic alterations with the primary tumor and originate from cells that branched off early during tumorigenesis [4]. These authors analyzed 252 TCGA samples and 23 paired GBM samples with the goal to better elucidate the intratumoral clonal composition of primary GBM, and to reveal how GBM responds to therapeutic interventions. Two mutational clusters were detected, namely the clonal cluster with mutations from before sequential malignant transformation, which mutations were present in all tumor cells; and the subclonal cluster with mutations that occured later during the tumor expansion and branching evolution, which mutations were present only in a subset of the tumor cells. The distribution of the mutations was 67.9% clonal, 29.8% subclonal, and 2.3% could not be classified. The majority of TP53 and PI3KCA/PI3KR1 mutations (90.5%) were clonal. This study revealed that most affected genes and pathways affect cell cycle control, DNA damage response, cell death and differentation that may all underlie gliomagenesis. The TP53 mutant recurrent GBMs showed an increase in subclonal mutation frequency compared to wild-type TP53 recurrent GBMs, which suggests an association between TP53 mutation and subclonal tumor evolution [4]. This observation may be related to the known involvement of TP53 in tolerance to DNA damage or apoptosis suppression [44, 45]. *Kim H.* et al [4] also found a linear correlation between clonal mutations and age. However, the subclonal tumor group showed relatively more favorable event-free survival, than the clonal tumor group, which may be explained by the absence of a dominant aggressive clone [4].

Given the size of the human diploid genome and the vast potential of the acquisition of mutations suggest that clonal mutations might be acquired prior to gliomagenesis over the life span of an individual [46]. A recent paper describing a GBM mouse model, however, underscores that the more differentiated a cell is, the less susceptibility for new mutations it has [47]. This elegant study strongly supports the hypothesis that pathogenic mutations in neurogen stem cells or in lineage progenitors initiate gliomagenesis and growth.

### Treatment-Induced Genomic Effects in Recurrent GBM

As mentioned above, the standard care for patients with newly diagnosed GBM includes surgical tumor removal and radio-chemotherapy, however, inevitably every tumor recurs [3, 48]. Recurrent tumors are less sensitive to therapy than the original tumors, and in most cases, invade functional brain areas, preventing a second surgical resection [5]. At present, it is unknown whether the primary reason for recurrence is lingering malignant cells, de novo clonal expansions or clonal selection under pressure from adjuvant radiation and chemotherapeutic treatments [49, 50]. There have been a few studies focusing on treatment effects in GBM where observations are available through sequential recurrences from the diagnosis to the patient’s death. *Nickel* et al. [51] analyzed a patient’s longitudinal tumor DNA samples by NGS, focusing on intratumor heterogeneity and the mutational differences between the primary tumor and recurrences. The patient was a 69 years old male. His treatment for the primary tumor involved surgical resection followed by radiotherapy and TMZ chemotherapy. At the first recurrence, he received the same treatment supplemented with thalidomine and bevacizumab, while at the second recurrence only surgical resection was applied. Prior to treatment, a PTEN mutation was noted in approximately half of the tumor cells, which likely acted as a driver mutation in the primary tumor. A small subset of cells also harbored PIK3CA mutation. The initial round of surgery, chemotherapy, and radiation did not eradicate the cells with the PTEN driver mutation, yet it was absent from the tumor at the second recurrence. In addition, the PIK3CA mutation harboring subclonal population acquired a „hypermutator” phenotype [51]. This observation is consistent with the results of some subsequent studies suggesting a potential hypermutator effect of TMZ-chemotherapy in low-grade gliomas and GBMs. However, following TMZ treatment hypermutation rarely develops in isocitrate dehydrogenase 1 (IDH1)-wild-type primary GBMs, indicating low risk for TMZ-induced hypermutation in these tumors under standard treatment. In contrast, the common IDH1 (R132H) mutation is known to induce a hypermethylation phenotype in gliomas by silencing MGMT (and other mismatch repair [MMR] genes) thus sensitizing the tumor to TMZ-induced mutagenesis [4, 16, 20, 52].

In another study, genomic DNA of 21 patients with primary and recurrent GBM were analyzed [21]. Three patients underwent only surgery without chemo- or radiation therapy. Analyzing the dynamic mutational profiles in the paired samples revealed evidence for therapy-mediated selection pressure in treated patients. These evidence included 1) decreased variant burden in recurrent tumors, 2) the neutral evolutionary pattern in untreated tumors shifted to non-neutral pattern in recurrent tumors after treatment, and 3) one recurrent tumor (out of the 18 TMZ-treated patients / tumors) showed a TMZ-induced hypermutator phenotype [21]. This latter observation suggesting a rarity of TMZ-induced hypermutation at recurrence is consistent with previous studies [4].

*Erson-Omay* et al. [53] treated and analyzed a single patient from diagnosis through double recurrences, and assessed the frequency of genomic events detected in this patient and in 110 exome- and whole-genome-sequenced specimens in the Yale-Glioma cohort. In the initial index patient’s tumor, WES analysis revealed amplification of chromosome 7 and deletion of chromosome 10 and focal deletion of CDKN2A locus on chromosome 9 along with an activating ectodomain EGFR A289V mutation, suggesting that the tumor cells had undergone chromothripsis. Chromothripsis is a sudden event with complex genomic rearrangements catastrophic for the harboring cell [54]. Based on the Yale-Glioma cohort, chromotripsis may be a frequent event in GBM. The patient in the *Erson-Omay* et al study [53] was enrolled in a clinical trial with a receptor tyrosine kinase inhibitor, vandetanib, beside the standard TMZ treatment. Despite all efforts, the disease progressed and a second gross total resection was carried out. WES analysis of the recurrent tumor showed a loss of tumor cells harboring the activating EGFR A289V mutation, most likely due to the targeted anti-EGFR therapy. However, this treatment had no impact on the high EGFR ploidy. After the second recurrence, DNA analyses showed double-minutes known to be resistant to targeted therapies [55, 56]. However, this tumor also harbored a hypermutator phenotype, involving the *MSH6* gene and the MMR mechanism, allowing to design a new therapeutic strategy. Numerous reports pointed out that hypermutated tumors including endometrial, gastric, colorectal and small bowel cancers are susceptible to immune checkpoint inhibitors [57]. Based on these observations, the above patient with GBM was started on hydroxyurea and immune checkpoint inhibitor, pembrolizumab, and he lived for 5 years after the diagnosis [53]. While immune checkpoint inhibitors are now part of the standard treatment protocols in several solid tumors (e.g. melanoma, non-small cell cancer lung cancer, etc.…), systematic testing of these agents in GBM was only recently reported. The Ivy Foundation Early Phase Clinical Trials Consortium study patients were randomized to neoadjuvant pembrolizumab (a programmed cell death protein 1 [PD-1] - specific monoclonal antibody) before surgery and pembrolizumab adjuvant therapy after surgery. Patients who received neoadjuvant pembrolizumab before surgery and continued on pembrolizumab after surgery had longer overall survival than patients who received adjuvant, post-surgical PD-1 blockade alone. Neoadjuvant pemrolizumab was associated with upregulation of T cell– and interferon-γ-related gene expression, but downregulation of cell-cycle-related gene expression in the tumor. The neoadjuvant pembrolizumab therapy enhanced both the local and systemic antitumor immune response [58]. In the study by *Zhao* et al [59], genomic and transcriptomic analyses of the tumors revealed an enrichment of PTEN mutations associated with immunosuppressive gene profiles in nonresponders, whereas an enrichment of MAPK pathway alterations was detected in responders. Single-cell RNA sequencing of one PTEN-mutant tumor of a non-responder showed the association of the immunosuppressive signature (T regulatory cells, macrophages, microglia, neutrophils) with CD44 + tumor cells involved in invasion. Analyses of clonal evolution of mutations in a few responders and nonresponders suggested that neoantigenic mutations were lost, while genes associated with immunosuppression were enriched following therapy with PD-1 inhibitors. *Schalper* et al [60] tested a presurgical nivolumab (another PD-1 specific antibody) followed by postsurgical nivolumab until disease progression. Tumor tissues pre- and post-nivolumab dosing and from patients without nivolumab were analyzed for changes in the tumor immune microenvironment. Neoadjuvant nivolumab induced an increased expression of chemokines along with enhanced immune cell infiltration and diversity of tumor-infiltrating T lymphocyte clonality. While impressive effects were observed at the cellular and molecular levels, clinical benefits of these three studies were modest, and only a few individual patients showed longer survival.

Taken together, the above studies suggest that TMZ treatment can impact tumor heterogeneity by reducing the diversity of tumor subclones (allowing the resistant sublones to become dominant at relapse), shift the neutral mutational dynamics towards non-neutral patters and in rare cases, increases the mutation burden (hypermutator phenotype). Emerging evidence supports that GBMs undergo evolutionary changes even in the absence of any therapy. However, the selective pressure exerted by radio-chemotherapies and targeted therapies without doubt alter the molecular composition of these tumors [61]. Immune checkpoint inhibitors although initially beneficially impacted the molecular profiles of glioblastoma, the evolution of mutational profiles and the immune suppressive changes in the tumor microenvironment annulled the early benefits eventually leading to tumor recurrence.

### Transcriptome Studies

Following the pivotal study of cross-sectional primary GBM specimens by Verhaak et al. [9], a number of elegant transcriptome analyses were published focusing on sequential GBM sampes. *Li* et al [62] investigated the transcriptome of 88 primary and 22 recurrent GBMs to define the distribution (heterogeneity) of molecular subtypes and gene signatures. Differences were detected in the distribution of the CL subtype in the recurrent (22%) and primary GBMs (36%), in the frequency of IDH1 mutations that were nearly twice as high in recurrent than in primary tumors, and in the frequency of TP53 mutations when PN recurrences (20%) and CL recurrences (80%) were compared. Furthermore, gene set enrichment analyses revealed that chromatine fracture, repair and remodeling gene sets were enriched in recurrent GBM coinciding with biological progression of the recurrent tumors [62]. Another interesting study focused on the interactions between the tumor and its microenvironment [10]. These authors compared the transcriptome profiles of 596 single glioma cells from 8 matching primary and recurrent gliomas, along with 124 additional glioma pairs from other datasets. Transcriptome profiling of tumor samples is a commonly used technique for interrogating pathway functionality and phenotype-based classification, however, the impact of tumor microenvironment may obscure the true transcriptional profile and the signaling activity in the tumor [63]. The study of *Wang Q.* et al [10] identified in their samples the TCGA-proposed transcriptional glioma subtypes, PN, CL and MES [9] however, the NE subtype was identified as normal cell contamination in the tumor sample. In the MES GBM subtype, the presence of tumor-associated glial and microglial cells was quite remarkable confirming that a macrophage/microglia-rich microenvironment shapes the MES glioma phenotype. The data also showed that neurofibromin (NF1) deactivation (characteristic of the MES phenotype) results in the attraction of macrophage/microglia, suggesting that there is a two-way interaction between tumor cells and microenvironment. The longitudinal transcriptome analysis showed that the gene expression-based subtype is retained in 55% of the cases [10], somewhat conflicting with another observation that two-thirds of primary GBMs exhibit different transcriptional subtypes at diagnoses and relapse [19]. Previously Verhaak et al. [9] suggested that three quarters of the tumors did not change class at recurrence [9], though this observation was based on a very small cohort (Murat dataset [64]). As both [10, 19] worked with large cohorts of specimens, the differences between their results indicate that transcriptome analyses may lead to dissimilar results depending on when and from where the samples are taken.

Finally, a few studies used single cell RNA-sequencing (scRNA-seq) to examine mutational differences between initial and recurrent GBMs [27, 28, 65]. Using scRNA-seq, *Patel* et al. [27] observed extensive ITH at transcriptional level. The authors also established GSC cultures to examine stemness signature, which subsequently applied to the single-cell transcriptional profiles revealed a stemness gradient in tumors. The expression of a number of transcription factors (NF1A, NF1B) previously implicated in tumor propagation and neural stem cell self-renewal, also significantly correlated with stemness gradient. In another study *Chen* et al [65] identified 3 relapse–specific homozygous missense mutations in three independent genes involved in RAS/GEF GTP-dependent signaling, which pathway is known to be involved in GBM pathogenesis [65, 66]. These studies highlighted that scRNA-seq is an approach suitable to gain a deeper insight into ITH and clonal evolution, while identifies defects in transcriptional programs related to oncogenic signaling and proliferation response.

### GBM Epigenome Studies

The most widely studied epigenetic mechanism in GBM is DNA CpG methylation. CpG island hypermethylation and through that gene silencing is a hallmark of human cancers, but certain sets of genes may get hypomethylated and thus overexpressed. DNA CpG methylation profiles have been used as biomarkers for early detection of tumors in blood or body fluids, or predicting prognosis or treatment response and to monitor cancer recurrence [67]. As reviewed above, most research studies on tumor heterogeneity in GBM involved genomic and transcriptomic analyses [4, 8–10, 19, 20, 27, 43, 68]. DNA methylation microarrays and sequencing of bisulfite converted tumor DNA identified characteristic differences in the methylation profiles of cross-sectional and sequential GBM specimens, while also revealed ITH [11, 69–71].

The most extensively investigated epigenetic mark in GBM is the methylation of the MGMT gene promoter, which is an independent prognostic factor for favorable tumor biology and a beneficial predictor of response to TMZ and radiotherapy [22]. Another important achievment in GBM epigenomics is the identification of glioma-CpG island methylathor phenotype (G-CIMP), which has provided the basis for subsequent epigenomic studies [11, 24] focused on epigenetic differences to classify diffuse IDH mutant and IDH-wild-type gliomas into further molecular subgroups with characteristic patient outcome. Analyses of the DNA methylation profiles from 200 tumors of 77 patients were carried out to elucidate the epigenome-based signs of malignant transformation of initially lower grade gliomas. The initially G-CIMP high turned out to carry the worst prognosis, and the capability to recur as a more aggressive G-CIMP-low tumor, which can mimic an IDH-wild-type and stem cell-like GBM at recurrence. Since histopathological grading at first diagnosis is unable to predict phenotype changes, identification of this subgroup has crucial clinical implications for the assessment and therapeutic management of aggressive low grade gliomas at risk for malignant recurrences [24]. Others examined non-glioma CpG island methylator phenotype (non-G-CIMP) tumors, and found that a subset of gene promoter hypomethylation (for example TP73, TERT) leads to up-regulation of alternate transcripts with potential oncogenic consequences [23]. The most complex and forward-looking study recently carried out by *Klughammer* et al [25] highlights the importance and broad applicability of epigenetic studies. From a technical perspective, this study was facilitated by an optimized reduced representation bisulfite sequencing (RRBS) technique suitable to study FFPE specimens. The fact, that the authors were able to identify transcriptional subtypes based on RRBS data shows the utility of this approach for molecular classification. Therefore, the RRBS-based bisulfite sequencing may circumvent the dependence on high-quality RNA in transcriptional subtyping. This study also addressed whether or not the RRBS profiles (complemented with detailed histopathological characterization of tumors) capture relevant aspects of tumor microenvironment. This part of the study revealed significant differences in immune cell infiltration among the three transcripctional subtypes. The highest number of immune cells was found in tumors of MES subtype, consistent with previous data [10]. DNA methylation well differentiated between samples with high and low percentage of necrosis in histopathology, and with high versus low levels of specific immune cell infiltrates and CD8-positive cells, suggesting that DNA methylation data can be used to infer immune cell infiltration [25]. From the aspect of tumor evolution, patient-specific DNA methylation profiles were largely retained, but substantial inter- and intratumor heterogeneity was identified, although without clear trend for higher or lower heterogeneity in primary and recurrent samples. Biological pathway analyses showed during progression an enrichment for genes that gained methylation among those involved in neuronal development and apoptosis, while genes whose promoters lost methylation were enriched in the Wnt signaling pathway and T cell activation [25]. Aberrant activation of the Wnt signaling pathway has been linked to stemness, invasiveness, angiogenesis and therapeutic resistance in cancer [72] and is also known to play important roles in GBM [29]. The above epigenomic studies reflect advantages of the approach, most importantly representing a proxy to RNA-based analyses when using clinical FFPE specimens, but with the cost of somewhat reduced resolution. The information gained by these studies not only supplements data revealed by other OMICS methods, but also allows us to gain a more complex picture regarding molecular characteristics, dynamics and potential therapeutical targets in GBM.

## Conclusions (Fig. 1)

The longitudinal studies briefly reviewed here have widened our understanding of GBM development and progression, however, several questions remained unanswered. We highlighted some important findings from IHC studies that revealed differences in protein expression patterns in primary and recurrent GBM, while also noted the limitations of such a low resolution, expression based evaluation method [33–35]. Subsequently, we surveyed the results of high-resolution genome-wide analyses in sequential GBM specimens, and presented the observed changes in the genomic, transcriptomic and epigenomic profiles over time as well as pointed to some treatment-induced effects on these profiles. These observations allow us to draw some conclusions on the evolution, dynamics and heterogeneity of the disease. The finding that the distally (in different microenvironment) recurred tumors share only a relatively low percentage of initial tumor mutations, in contrast to the locally (in relatively similar microenvironment) recurred ones would suggest that recurrence at a distant location involves clonal selection and genomic divergence, while local recurrence involves some degree of linearity [20]. However, the observation that a dominant clone in a recurrent tumor is generally not necessarily the linear descendant of the dominant clone of the initial tumor suggests that genomic alterations may independently arose in different founder cells [19]. It is also important to note that many tumors exhibit different transcriptional subtypes upon recurrence, and a given GBM specimen may also simultaneously harbor more than one molecular subgroup, even though detection of single molecular subgroups both in cross-sectional and longitudinal specimens have been noted [8, 27, 28]. Determination of the DNA CpG methylation patterns genome-wide allows us to infer the transcriptional subtypes of GBM even in FFPE specimens, and provides further insight into dynamics and pathways of molecular changes as well as of ITH [25]. Altogether, these observations suggest that the development of GBM over time is highly idiosyncratic, and follows different patterns and dynamics of molecular evolution. The accumulation of molecular changes may be influenced by prior mutations (e.g. IDH1, TP53, MMR genes) and gene expression profiles (DNA CpG hypomethylation and hypermethylation), the impact of microenvironmental changes (soluble molecules, cell-cell interactions, shifts in immune modulation) and treatment effects (traditional TMZ and irradiation, immune checkpoint inhibitors, and emerging other medications). Thus far, however, all approaches unequivocally establish that molecular profiles and dynamics of GBMs are extremely divers even though with some identifiable unifying features (e.g. existence of molecular subgroups). Therefore, success in treatment may only be expected from complex, likely individually adjusted therapeutic strategies, which need to be based on deep molecular profiling.

**Fig. 1:**
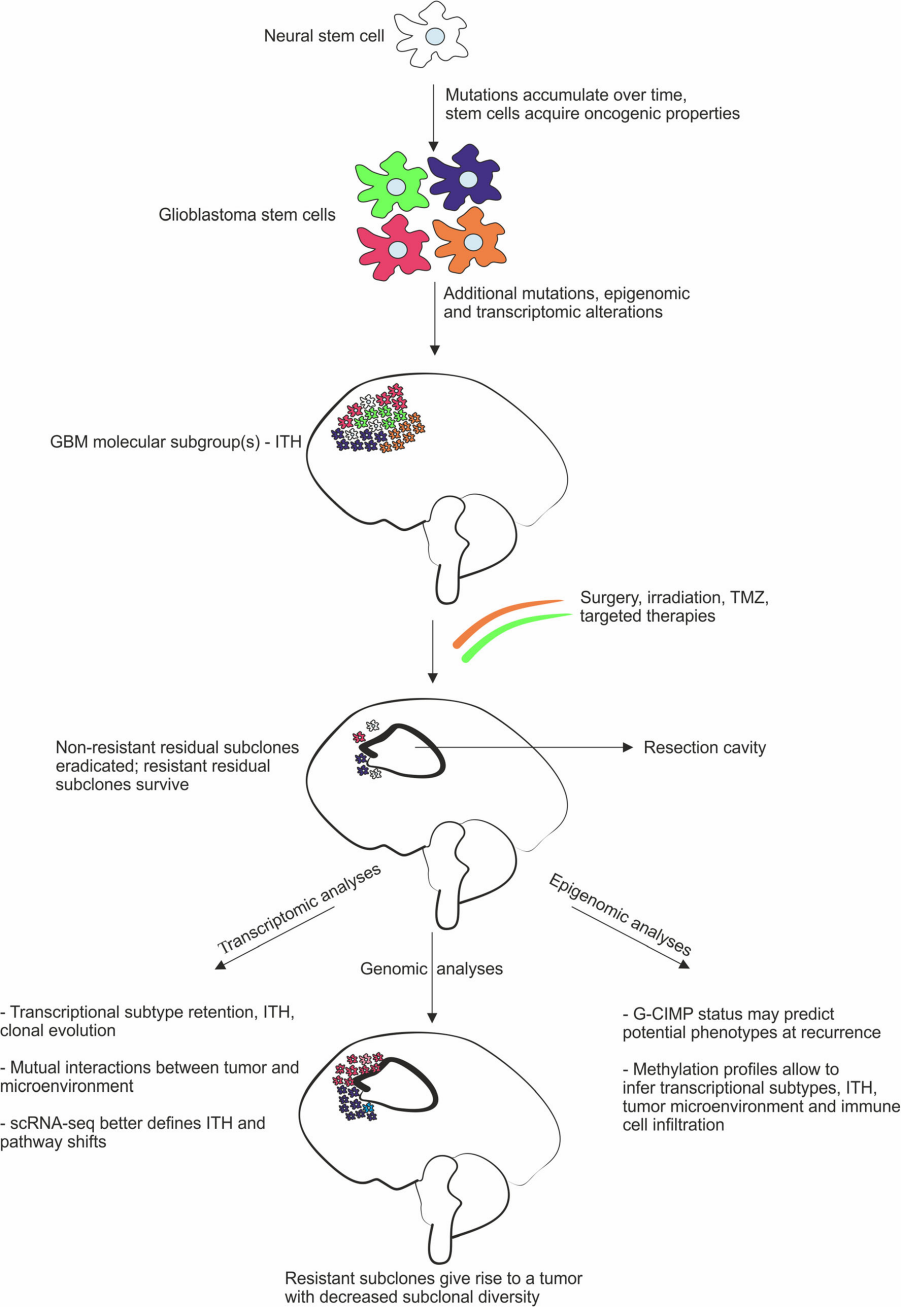
Molecular evolution of glioblastoma. The figure synthesizes main points of genomic, transcriptomic and epigenomic studies on longitudinal glioblastoma specimens and reflects the complexity of possible outcomes
